# MELK-Dependent FOXM1 Phosphorylation is Essential for Proliferation of Glioma Stem Cells

**DOI:** 10.1002/stem.1358

**Published:** 2013-02-13

**Authors:** Kaushal Joshi, Yeshavanth Banasavadi-Siddegowda, Xiaokui Mo, Sung-Hak Kim, Ping Mao, Cenk Kig, Diana Nardini, Robert W Sobol, Lionel ML Chow, Harley I Kornblum, Ronald Waclaw, Monique Beullens, Ichiro Nakano

**Affiliations:** a Department of Neurological Surgery, The James Comprehensive Cancer Center, The Ohio State UniversityColumbus, Ohio, USA; b Center for Biostatistics, The Ohio State UniversityColumbus, Ohio, USA; c Department of Psychiatry, University of California Los AngelesLos Angeles, California, USA; d Department of Molecular and Medical Pharmacology, University of California Los AngelesLos Angeles, California, USA; e Cancer and Blood Diseases Institute at Cincinnati Children's Hospital Medical CenterCincinnati, Ohio, USA; f Division of Experimental Hematology and Cancer Biology at Cincinnati Children's Hospital Medical CenterCincinnati, Ohio, USA; g Department of Pharmacology & Chemical Biology, University of Pittsburgh Cancer InstitutePittsburgh, Pennsylvania, USA; h Department of Human Genetics, University of Pittsburgh Cancer InstitutePittsburgh, Pennsylvania, USA; i Laboratory of Biosignaling & Therapeutics, Department of Cellular and Molecular MedicineKULeuven, Leuven, Belgium

**Keywords:** Neural stem cell, Cancer stem cell, Glioblastoma, Glioblastoma stem cell, PLK1

## Abstract

Glioblastoma multiforme (GBM) is a life-threatening brain tumor. Accumulating evidence suggests that eradication of glioma stem-like cells (GSCs) in GBM is essential to achieve cure. The transcription factor FOXM1 has recently gained attention as a master regulator of mitotic progression of cancer cells in various organs. Here, we demonstrate that FOXM1 forms a protein complex with the mitotic kinase MELK in GSCs, leading to phosphorylation and activation of FOXM1 in a MELK kinase-dependent manner. This MELK-dependent activation of *FOXM1* results in a subsequent increase in mitotic regulatory genes in GSCs. MELK-driven *FOXM1* activation is regulated by the binding and subsequent trans-phosphorylation of FOXM1 by another kinase PLK1. Using mouse neural progenitor cells (NPCs), we found that transgenic expression of FOXM1 enhances, while siRNA-mediated gene silencing diminishes neurosphere formation, suggesting that FOXM1 is required for NPC growth. During tumorigenesis, FOXM1 expression sequentially increases as cells progress from NPCs, to pretumorigenic progenitors and GSCs. The antibiotic Siomycin A disrupts MELK-mediated FOXM1 signaling with a greater sensitivity in GSC compared to neural stem cell. Treatment with the first-line chemotherapy agent for GBM, Temozolomide, paradoxically enriches for both FOXM1 (+) and MELK (+) cells in GBM cells, and addition of Siomycin A to Temozolomide treatment in mice harboring GSC-derived intracranial tumors enhances the effects of the latter. Collectively, our data indicate that FOXM1 signaling through its direct interaction with MELK regulates key mitotic genes in GSCs in a PLK1-dependent manner and thus, this protein complex is a potential therapeutic target for GBM. Stem Cells *2013;31:1051–1063*

## INTRODUCTION

Glioblastoma multiforme (GBM) is the most common primary malignant brain tumor and is highly aggressive and therapy-resistant [1–3]. Even patients with well-demarcated tumors in noneloquent areas that allow for gross-total resection at surgery and who respond well to first-line chemotherapies and radiotherapies frequently do not escape from subsequent recurrence and ultimately die from disease progression. Therefore, there is an urgent need to develop novel therapies to effectively target resistant GBM cells. GBM is composed of mixed tumor cell populations including tumor cells with stem cell properties, termed glioma stem-like cells (GSCs) [4, 5] Accumulating evidence suggests that stem cell properties of GSCs contribute to therapeutic resistance in GBM. Tumor stem cells are one, if not the only, cellular target in GBM, and therapeutic development targeting this subset of tumor cells may improve patient survival [6].

The transcription factor, FOXM1 is a member of the Forkhead box (FOX) family that consists of more than 50 mammalian proteins with shared homology in the winged helix DNA binding domain. Many genes within this family play critical roles in cell cycle progression and cell fate decision. Initial studies of FoxM1 characterized its role in liver development [7]. Intriguingly, FoxM1 knockout mice fail to form hepatocellular carcinoma in a carcinogenic induction model, suggesting that FoxM1 is necessary for tumor initiation in the liver [8]. Accumulated evidence suggests that FOXM1 is a proto-oncogene with elevated expression in a number of human cancers such as liver, ovarian, breast, prostate, colon, and brain tumors including GBM. Downstream target pathways of FOXM1 include vascular endothelial growth factor (VEGF), matrix metalloproteinase-2 (MMP-2), and β-catenin [9–11], each of which promotes tumor formation and progression. FOXM1 enhances cancer stem cell self-renewal through direct binding to β-catenin inducing nuclear localization and transcriptional activity [10]. Taken together, abrogation of FOXM1 signaling may provide multidirectional approaches for controlling cancers including GBM.

Maternal embryonic leucine-zipper kinase (MELK) is a serine/threonine kinase and is abundantly expressed in GBM and various other cancers. Previously, we found that MELK is highly expressed in GSCs derived from GBM samples and its mRNA expression is inversely correlated with survival in GBM patients [12]. In addition, small inhibitory RNA (siRNA)-mediated MELK abrogation induces GSC apoptosis in vitro with less inhibitory effects on normal neural progenitor cells (NPCs) [12]. A recent study using a murine breast cancer initiation model indicated that ablation of Melk eliminates tumorigenesis in vivo; thus MELK is likely a therapeutic target not only for GBM but also for other cancers [13].

Prior studies have suggested that both FOXM1 and MELK play essential roles in cell cycle progression, cancer cell growth, and maintenance of stem cell state of GBM. Furthermore, MELK and FOXM1 are highly coexpressed and coregulated in GBM suggesting that they are functionally related. Therefore, we sought to test the hypothesis that MELK and FOXM1 directly interact to regulate proliferation of GSC [14]. In this study, we interrogated the physical interaction of FOXM1 and MELK, as well as the functional roles of FOXM1-MELK signaling in GSCs. Here, we describe novel mechanistic evidence that FOXM1 cooperates with MELK to regulate the mitotic transcriptome in GSCs.

## MATERIALS AND METHODS

### Human Specimens and Tissue Culture

Neurospheres (NS) derived from 10 GBM samples and 1 fetal brain were used in this study, as described previously [12, 15-17]. Regarding the nine samples collected at The Ohio State University (OSU), surgery was performed by I. Nakano and E.A. Chiocca in the Department of Neurological Surgery, and surgical specimens were processed for research under approved protocols (IRB Number 2005C0075). GBM157 was established in Dr. Kornblum's laboratory at UCLA, as described previously [18]. Established GSC cultures were cultured in defined medium containing Dulbecco's modified Eagle's medium (DMEM)/F12/Glutamax (Invitrogen, Carlsbad, CA, http://www.invitrogen.com) supplemented with 2% B27 supplements (Invitrogen) and 20 ng/ml epidermal growth factor (EGF) and fibroblast growth factor-2 (PeproTech, Rocky Hill, NJ, http://www.peprotech.com), as previously reported [19]. Mouse neural progenitors were cultured from the subventricular zone (SVZ) of the day 17 embryos, as described previously [17]. 293T cells were obtained from the American Type Culture Collection and cultured in DMEM (Invitrogen) supplemented with 10% fetal calf serum (Sigma Aldrich, St. Louis, MO, http://www.sigmaaldrich.com). For more detailed methods, see Supporting Information.

### Vectors

All the FOXM1 vectors (wild-type and mutants, S715A, S724A, double mutant S715/724A,S678A, T596A, TSAA,EE and the reporter 6x FoxM1) and PLK1 (wild-type and dominant negative) were kindly provided by Dr. Zheng Fu of the Virginia Commonwealth University. All MELK vectors were described previously [20].

### Phosphorylation Assay

FOXM1 cDNA was inserted in enhanced green fluorescent protein (pEGFP)-C1 (Clontech, Palo Alto, CA, http://www.clontech.com) for mammalian cell expression and pet16b (Novagen) for bacterial expression. *Flag*-MELK and pet16b-MELK1-340 were described previously [20]. HEK293 cells were transiently transfected with the indicated plasmids, and cell lysis was performed as described [20]. The complexes were trapped with GFP-trap beads (Chromotek, Germany, http://www.chromotek.com) and analyzed for the presence of *Flag*-tag fused MELK with *Flag* antibody (Stratagene, La Jolla, CA, http://www.stratagene.com). His-MELK1-340, His-FOXM1, and His-cyclinA-CDK2 were expressed in bacteria and purified as described previously [20, 21]. His-FOXM1 was phosphorylated in vitro by His-MELK1-340, His-MELK1-340D150A, or His-cyclinA-CDK2 for 1 hour at 30°C in a buffer containing 25 mM Tris at pH 7.5, 0.1 mM [γ-^32^P] ATP, 2 mM magnesium acetate, and 20 mM dithiothreitol (DTT).

### Gene Expression Omnibus Profile and TCGA Data

Affymetrix Human Genome U133A Array was performed as described previously. The data have been submitted to the GEO database accession number GDS1815 [22, 23]. *MELK* and *FOXM1* expression (Affymetrix Human Genome U133A Array) data were downloaded from the GDS1815 dataset and analyzed for grade III glioma and GBM. The Cancer Genome Atlas (TCGA) data are available through the TCGA Data Portal at http://tcga-data.nci.nih.gov [24].

### Drug Treatment

Siomycin A was obtained through the Developmental Therapeutics Program NCI/NIH. Siomycin A and ON-01910 (PLK1 inhibitor-selleck chemical Inc., Houston, TX, USA, http://www.selleckchem.com) stock solution was prepared using dimethyl sulfoxide (DMSO) (Sigma Aldrich). In each experiment, DMSO alone was used in control samples at a concentration in between 0.1% and 1% and identical to the concentrations of DMSO that was used in drug-treated cells in each experiment. We confirmed that the growth of the cells we used in this study is not significantly affected by DMSO at 1% or lower [18].

### Statistics

Quantitative data are presented as means ± SD, unless noted otherwise in the figure legend. The numbers of replicates are noted in the figure or legends. Comparison of mean values between multiple groups was evaluated by an ANOVA followed by Tukey's test. When multiple comparisons were involved, Holm's method was used to adjust the multiplicities to control the type I error rate that was less than 0.05 [25]. Spearman correlation coefficients (ρ) were used to assess the correlation between *FOXM1* and *MELK* expression in the GDS1815 microarray data and TCGA dataset [26]. Log-rank tests were used to compare the survival probabilities between groups in the xenograft mouse experiment. Comparison of mean values between two groups was evaluated by χ^2^ test or *t* test. Log-rank analysis is used to determine statistical significance of Kaplan Meier survival curve. For all statistical methods, a *p*-value less than .05 was considered significant. For more detailed information, see Supporting Information.

## RESULTS

### FoxM1 Expression Is Restricted to NPCs in the Mouse Brain

**Figure 1:**
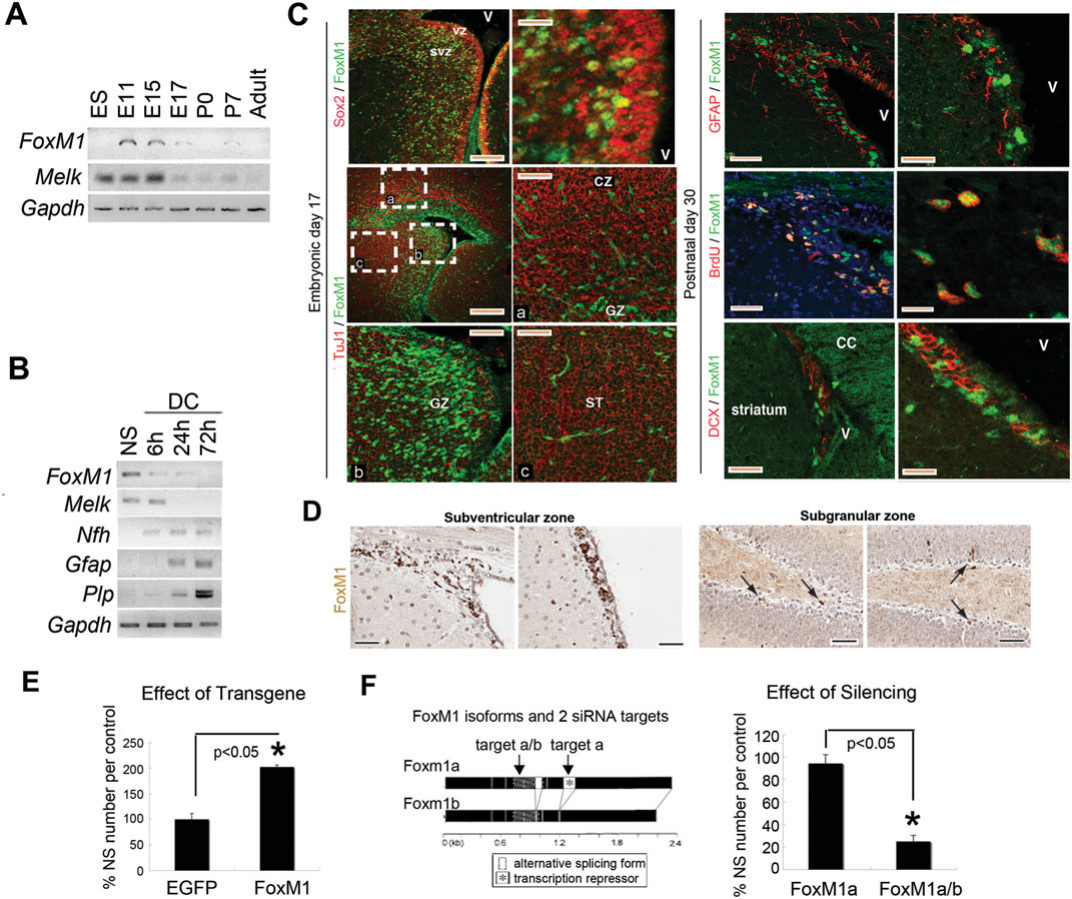
FoxM1 expression is restricted in neural progenitor cells in the mouse brain. **(A):** Reverse transcription polymerase chain reaction (RT-PCR) analysis for *FoxM1* and *Melk* expression during brain development. **(B):** RT-PCR analysis for *FoxM1* and *Melk* in neural progenitor cells with or without differentiation. Expression is compared with the differentiation markers for neurons (*neurofilament heavy chain* [*Nfh*]), astrocytes (*Gfap*), and oligodendrocytes (*proteolipid protein* [*Plp*]). For (A) and (B), *Gapdh* is used as internal control. **(C):** Left panels, top two images indicate immunohistochemistry for FoxM1 (green) and Sox2 (red) in mouse brains of embryonic day 17 (E17). Lower four pictures, immunofluorescence for FoxM1 (green) and Tuj1 (red) in mouse brains of E17. Original magnification is ×10 and ×40. Scale bars = 100 and 20 μm. (a) CZ, (b) GZ, (c) ST represents magnified pictures of FOXM1 (green) and Tuj-1 (red). Original magnification is ×10 and ×40. Scale bars = 100 and 20 μm. Right panels represent immunohistochemistry for FoxM1 (green), Gfap (red), BrdU (red), and Dcx in brains of the postnatal mouse day 30. Original magnification is ×20–×40. Scale bars = 50–20 μm, respectively. **(D):** Immunohistochemistry of FOXM1 (brown) in adult mouse brain at the SVZ (left panel) and at the subgranular zone (right panel). Original magnification is ×40. Scale bars = 20 μm. **(E):** Bar graph representing the relative number of NS formed from EGFP- or FoxM1-expressing NS derived from mouse E17 cerebral cortices. Asterisks (*) indicate statistical significance by *t* test. **(F):** Left panel exhibiting diagram of two different isoforms of FoxM1 protein. Right panel indicating the relative number of NS formed from FoxM1a or FoxM1a/b siRNA transfected NS derived from mouse E17 cerebral cortices. NS forming assay was performed in triplicate in 96-well plate and repeated three times independently. Abbreviations: BrdU, Bromodeoxyuridine; CC, corpus callosum, CZ, cortical zone; DCX, doublecortin; DC, differentiated cells; EGFP, enhanced green fluorescent protein; ES, embryonic stem cells; E, embryonic brain; GFAP, glial fibrillary acidic protein; GZ, germinal zone; NS, neurospheres; P, postnatal brain; SVZ, subventricular zone; ST, striatum; V, ventricle; VZ, ventricular zone.

First, we investigated *FoxM1* expression during brain ontogeny. Similar to *Melk* expression, *FoxM1* expression in the brain was predominantly detected during early and mid-embryonic periods with a dramatic decline between embryonic day 15 (E15) and E17 (Fig. 1A). Expression of *FoxM1* in the adult brain was below detectable levels by reverse transcription polymerase chain reaction (RT-PCR). We then examined *FoxM1* expression in neural progenitor cultures grown as NS derived from embryonic mouse cortices. *FoxM1* expression was markedly high in proliferating NS, whereas its expression levels declined dramatically in the prodifferentiation—as indicated by expression of neuronal and glial markers—conditions within 6 hours, the earliest time point studied (Fig. 1B). These data suggest that, similar to *Melk* expression, *FoxM1* is preferentially expressed in proliferating murine NPCs.

We then performed immunohistochemistry of FoxM1 with mouse embryonic and adult brains. FoxM1 protein expression was restricted to the germinal zone (GZ) of embryonic day 17 (E17) brains. The majority of FoxM1 (+) cells were not colocalized with neuronal marker TuJ1 and vice versa (Fig. 1C, left panels; Supporting Information Fig. S1). In contrast, we observed extensive overlap of FoxM1 (+) cells with Sox2 (+) cells in the GZ. In brains at the postnatal day 30 (P30), FoxM1 expression was restricted to the proliferative zones lining the ventricles and was not colocalized with glial fibrillary acidic protein (GFAP), suggesting that FoxM1 is unlikely to be expressed in differentiated astrocytes or type B neural stem cells (NSCs) (Fig. 1C, right panels). In addition, there was no detectable coexpression of FoxM1 and the neuroblast marker Dcx. On the other hand, FoxM1 was expressed by proliferating cells, as indicated by colocalization with BrdU. Taken together, these data suggest that the majority, if not all, FoxM1 (+) cells are rapidly proliferating type C progenitor cells in the SVZ. When FoxM1 expression was evaluated in the other neurogenic region, the subgranular zone (SGZ) of hippocampus, very few cells, if any, in the SGZ express FoxM1 at P30 (Fig. 1D).

### FoxM1 Regulates NPC In Vitro

To determine the function of FoxM1 in NPCs, we assessed the effects of transgenic expression and silencing of the active FoxM1 isoform b on NS derived from E17 cerebral cortices. These neural progenitors were transfected with either the expression vectors or double-stranded RNAs designed to be siRNA for FoxM1 (isoform b) or controls. For knockdown of FoxM1, we designed two control siRNAs that silence either nontarget sequence or the transcription repressor region of FoxM1 isoform a, that is not present in the translationally active form of FoxM1, isoform b (Fig. 1E) [27]. Overexpression of FoxM1 (isoform b) resulted in a twofold enhancement of NS formation (Fig. 1E; Supporting Information Fig. S2). Although siRNA-mediated silencing of the inactive FoxM1 (isoform a) did not alter the growth of NS compared with the nontarget control, siRNA directed against the common region of FoxM1 (isoforms a and b) resulted in a significant decrease in NS numbers (Fig. 1F, right panel; Supporting Information Fig. S2). Collectively, these data suggest that FoxM1 is essential for the growth of murine NPCs derived from embryonic brains in vitro.

### FoxM1 Expression Is Markedly Elevated During Gliomagenesis

**Figure 2:**
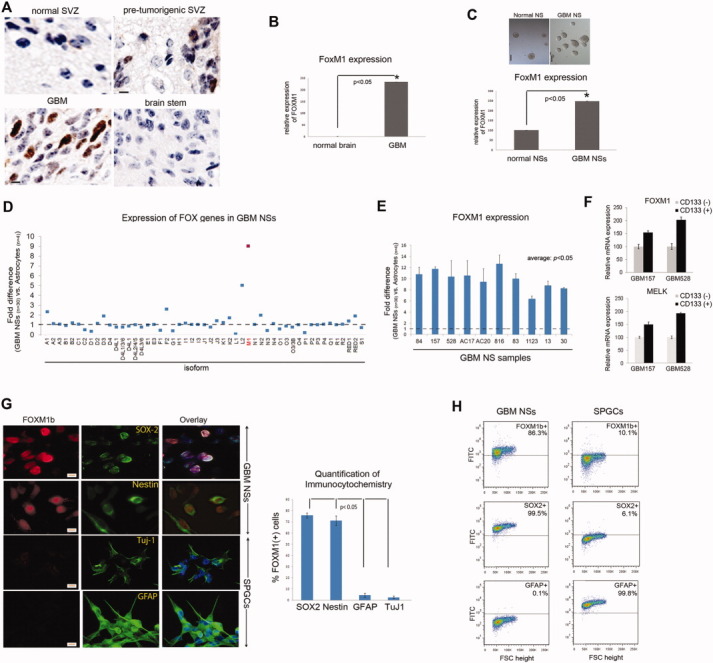
FoxM1 expression is markedly elevated during gliomagenesis. **(A):** Representative images of immunohistochemistry for FoxM1 (brown) in the SVZ of wild-type mice, SVZ of pretumorigenic Mut6 mice, and GBM-like tumors in cerebral cortex or normal areas in brain stem in Mut6 mice. Original magnification is ×10. Scale bars = 100 μm. Magnified images are shown below. Original magnification is ×40. Scale bars = 20 μm. **(B):** Quantitative reverse transcription polymerase chain reaction (qRT-PCR) analysis for *FoxM1* expression in GBM tissues in Mut6 mice compared to normal side brain tissues derived from the same mice. Asterisks (*) indicate statistical significance by *t* test. Experiment repeated in three Mut-6 mouse-derived GBM tissues. **(C):** Phase bright representative images of NS derived from either normal SVZ or GBM-like tumors developed in Mut6 mice. Original magnification: ×10. Scale bars = 100 μm. Lower panel indicates qRT-PCR data for FoxM1 expression in NS derived from GBM-like tumors (GBM NS) in Mut6 mice or normal SVZ (normal NS). Asterisk (*) indicates statistical significance by *t* test. Experiments were done in triplicate and repeated three times independently. **(D):** Microarray analysis of relative *FOX* genes family expression in GBM (*n* = 30) compared to normal astrocytes cultures (*n* = 4). Expression of astrocytes was normalized as one. Expression of *FOXM1* is shown in red. **(E):** Relative expression of *FOXM1* of 10 GSCs samples compared to normal astrocytes cultures. *FOXM1* expression of normal astrocytes was normalized as one. **(F):** Relative mRNA expression of FOXM1 and MELK in GBM157 and GBM528 CD133 (+) and CD133 (−) cells collected after cell sorting (*n* = 3). **(G):** Representative immunocytochemistry of GBM NS and GBM cells propagated in serum-containing medium (SPGCs) derived from GBM30. Cells were double-stained for FOXM1 (red) in combination with one of the following neural progenitor cell markers Nestin, SOX2 and differentiation markers TuJ1, GFAP (green). Hoechst dye is used for nuclear staining (blue). Original magnification: ×40. Scale bars = 20 μm. Right panel indicates of the proportions of FOXM1 positive cells coexpressing SOX2, Nestin, GFAP, and TuJ1 positive cells. For quantification, GBM30 sample were used and the experiment was repeated four times. **(H):** Flow cytometry analysis for the expression of FOXM1, SOX-2, and GFAP using GBM NSs and SPGCs, both of which are derived from GBM30. Abbreviations: FITC, fluorescein isothiocyanate; FSC, forward scatter; GFAP, glial fibrillary acidic protein; GBM, glioblastoma multiforme; NS, neurosphere; SPGC, serum-propagated GBM cells; SVZ, subventricular zone.

Accumulating evidence suggests that GSCs and their somatic counterparts, NPCs, share various fundamental signaling pathways to regulate their self-renewal and proliferation. We therefore sought to determine the role of FoxM1 in GBM-like tumors and GSCs in mice harboring conditional inactivation of the human GBM-relevant tumor suppressors p53, Nf1, and Pten [28]. In this model, Nestin promoter-driven Bromodeoxyuridine (BrdU)(+) cells along the ventricular wall are the cells of origin for the cortical GBM-like tumors formed in adult mice [28]. When we compared FoxM1 staining of the SVZ of wild-type mice and genetically engineered mutant GBM mouse brains, we observed sequential elevation of FoxM1 expression along the ventricular wall during the course of tumorigenesis (Fig. 2A; Supporting Information Fig. S3). When FoxM1 expression was compared between normal brain regions versus tumor areas of individual mice, tumors had more than 200-fold higher expression than normal brain regions (Fig. 2B). We then compared FoxM1 expression in NS derived from the SVZ of wild-type mice and GBM-like tumors. *FoxM1* mRNA levels were 2.5-fold greater in GBM NS than in normal NS, indicating that *FoxM1* expression is elevated in GSCs compared to NPCs in vitro (Fig. 2C) [29].

The *FOX* family transcription factors play a crucial role in organ development and cancer initiation and propagation in a context-dependent manner [30, 31]. To determine which members of the *FOX* gene family are upregulated in GSCs, we evaluated expression levels of all the *FOX* genes in the transcriptome microarray data derived from 30 patient-derived GSC samples as well as 4 normal astrocytes primary cultures (Fig. 2D). Among 51 FOX family members, *FOXM1* exhibited strikingly higher expression in GSCs in comparison to normal astrocytes, unlike any other *FOX* family members. In all of the tested 10 GSC samples, we observed 6–12-fold higher *FOXM1* expression by quantitative RT-PCR compared to astrocytes (Fig. 2E). CD133 expression on the cell surface is associated with cancers stem cells in some, if not all, GBM tumors [1, 4, 16, 32-34]. We further performed cell sorting by using CD133 marker and separated CD133 (+) and CD133 (−) cells for GBM157 and GBM528 GSCs. qRT-PCR demonstrated higher expression of *FOXM1* and *MELK* in CD133 (+) cells than CD133 (−) cells in both samples (Fig. 2F).

We then examined FOXM1 protein expression in human GSCs. In agreement with previous studies, immunohistochemistry of a human GBM specimen demonstrated strong immunoreactivity for FOXM1 in the nuclei of GBM cells (Supporting Information Fig. S4) [35]. Immunocytochemistry of dissociated GBM NS demonstrated that FOXM1 was colocalized with the stem cell-associated markers including Nestin and SOX2 but not with the differentiation markers, TuJ1 or GFAP (Fig. 2G). Protein expression analysis by flow cytometry also yielded similar results. Similar to the higher proportion of tumor cells expressing the NSC-associated protein SOX2 and in contrast to the lower proportion of tumor cells expressing the astrocytic marker GFAP in GSCs in serum-free conditions, FOXM1 (+) cells were predominantly detected in GSCs in serum-free medium (GBM NSs), when compared to GBM cells propagated in serum-containing medium (SPGCs) (Fig. 2H; Supporting Information Fig. S5). Subsequently, we analyzed *FOXM1* promoter activity in GSCs and non-GSCs in GBM30. GSCs manifested a significantly higher *FOXM1* promoter activity in comparison to SPGCs (Supporting Information Fig. S6). Taken together, FOXM1 expression and activity are substantially elevated in GSCs compared to SPGCs and normal cells in mouse and human brains.

### FOXM1 Is a Substrate for MELK

**Figure 3:**
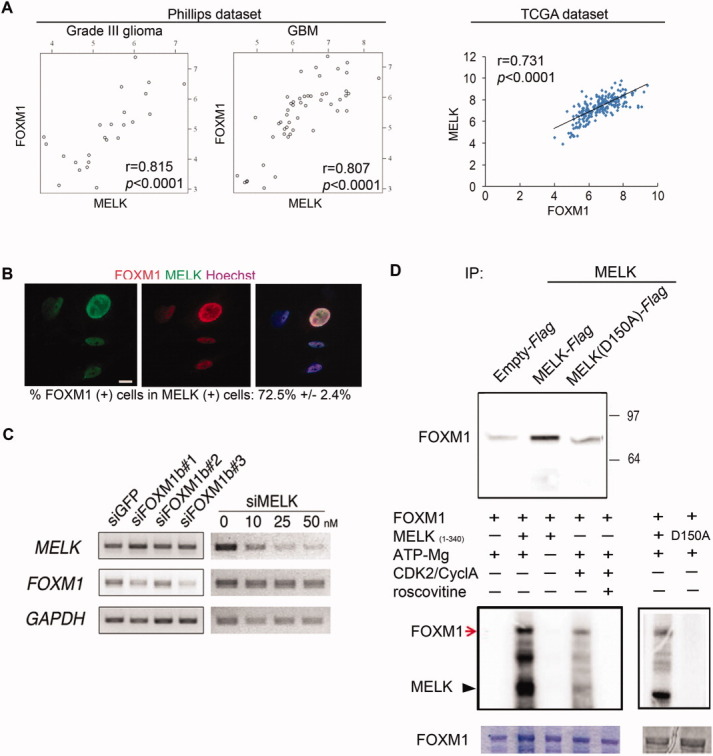
FOXM1 is a substrate for MELK. **(A):** Comparison of *MELK* and *FOXM1* expression profile (Affymetrix Human Genome U133A Array) indicates statistically significant correlation of the expression of these two genes in grade III glioma (left) (*n* = 24) and in GBM (middle) (*n* = 56). The Cancer Genome Atlas (TCGA) analysis of *MELK* and *FOXM1* expression profile in 218 GBM patient samples indicates statistical significance (right panel). **(B):** Representative images of immunocytochemistry with GBM30 neurospheres for FOXM1 (red), MELK (green). Hoechst dye for nuclear staining (blue). Original magnification: ×40. Scale bar = 20 μm. For quantification, GBM30 samples were used and the experiment was repeated four times. **(C):** Reverse transcription polymerase chain reaction (RT-PCR) analysis for *MELK* and *FOXM1* expression in GBM neurospheres treated with siRNA targeting three different sequences for FOXM1 (left). RT-PCR analysis for *MELK* and *FOXM1* expression in GBM neurospheres treated with different doses of siRNA targeting MELK (right) *n* = 5. **(D):** Upper panel: Overexpression of Empty-EGFP (control) + MELK-*Flag (Flag-MELK*), EGFP-FOXM1 + MELK-*Flag*, or EGFP-FOXM1 + MELK D150A-*Flag* plasmids in HEK293 cells are processed to GFP-trap followed by immunoblotting with anti-*Flag* antibody. Middle panel: Autoradiogram displaying in vitro phosphorylation of FOXM1 by the kinase domain of MELK (1–340). Lane 1: FOXM1 + ATP-Mg (no kinase); lanes 2 and 6: FOXM1 + ATP-Mg + MELK (1–340); lane 3: FOXM1 + MELK (1–340) (no ATP-Mg); lane 4: FOXM1 + ATP-Mg + CDK2/CyclA; lane 5: FOXM1 + ATP-Mg + CDK2/CyclA + roscovitine; lane 7: FOXM1+ ATP-Mg + MELK1–340(D150A). Lower panels: Coomassie staining of the samples subjected to autoradiography. Abbreviations: GBM, glioblastoma multiforme; IP, immunoprecipitation.

Prior studies have suggested that both FOXM1 and MELK play essential roles in the cell cycle progression, cancer cell growth, and maintenance of stem cell state of GBM [18, 20, 32, 35, 36]. Nonetheless, no studies have demonstrated a direct molecular interaction of MELK and FOXM1. We therefore sought to elucidate the potential molecular interaction of *FOXM1* and *MELK*. First, we used the TCGA array data set (GEO database accession number GDS1815) to determine the similarities of the expression profiles between *FOXM1* and *MELK* in high-grade glioma (Fig. 3A). Using 24 grade III glioma samples and 56 GBM samples, we found that *FOXM1* RNA expression in individual tumors was correlated with *MELK* expression (*p* < .001). Significant correlation was also observed in newly diagnosed and recurrent GBM patient samples at RNA levels (Supporting Information Fig. S7). We then sought to expand our analysis of their expression in GBM tissues through The Cancer Genome Atlas (TCGA), a publicly available repository which has accumulated comparative genomic hybridization, gene expression, and DNA methylation analyses for 218 GBM samples [24]. Using this database, coexpression of MELK in GBM tumor samples was confirmed (*p* < .0001) with FOXM1 expression. Immunocytochemistry with patient-derived GBM NS (GBM30) exhibited colocalization of FOXM1 with MELK within individual cells (72.5% ± 2.4% colocalization in Fig. 3B).

Since FOXM1 and MELK are expressed in the same cells, we next investigated whether levels of MELK influence FOXM1 expression and vice versa (Fig. 3C; Supporting Information Fig. S8). Both RT-PCR and Western blot demonstrated that MELK expression is not affected by FOXM1 transgenic expression or silencing. Likewise, RT-PCR analysis demonstrated that neither MELK overexpression nor knockdown affected FOXM1 expression. We then asked whether FOXM1 physically interacts with MELK. HEK 293 cells overexpressing both *Flag*-tagged MELK and the fusion protein of FOXM1 and EGFP or EGFP alone (control) were subjected to EGFP trap, followed by Western blot with MELK or *Flag* antibody (Fig. 3D, upper panel; Supporting Information Fig. S9). Cells co-overexpressing MELK and FOXM1 exhibited a stronger band for MELK than the control samples, implying a physical interaction between MELK and FOXM1 protein. This protein–protein interaction is likely dependent on MELK kinase activity, as the band intensity for the co-immunoprecipitation (IP) of the catalytically dead MELK mutant (D150A) was substantially diminished. Furthermore, we confirmed the direct interaction of these two proteins with an in vitro interaction study using recombinant purified FOXM1 and the purified N-terminal MELK-fragment (amino acid 1–340) but not with the C-terminal fragment (not shown).

We next asked whether recombinant MELK phosphorylates FOXM1 (Fig. 3D, lower panels). Purified recombinant FOXM1 was incubated with or without the purified kinase domain of MELK in an in vitro phosphorylation assay. In parallel, we used purified CDK2/cyclin A kinase, a known FOXM1 kinase, as a positive control [37]. The autoradiogram clearly shows in vitro phosphorylation of FOXM1 by MELK and CDK2/cyclinA. In addition, FOXM1 phosphorylation was not observed when incubated with kinase dead mutant MELK D150A. The same amount of FOXM1 was present in all conditions, as is shown on the corresponding Coomassie staining. Collectively, these data indicate that MELK directly interacts with FOXM1 and thereby, phosphorylates FOXM1 in a kinase dependent manner.

### *MELK* Activates *FOXM1* Transcriptional Activity Leading to Upregulation of Mitotic Gene Expression

**Figure 4:**
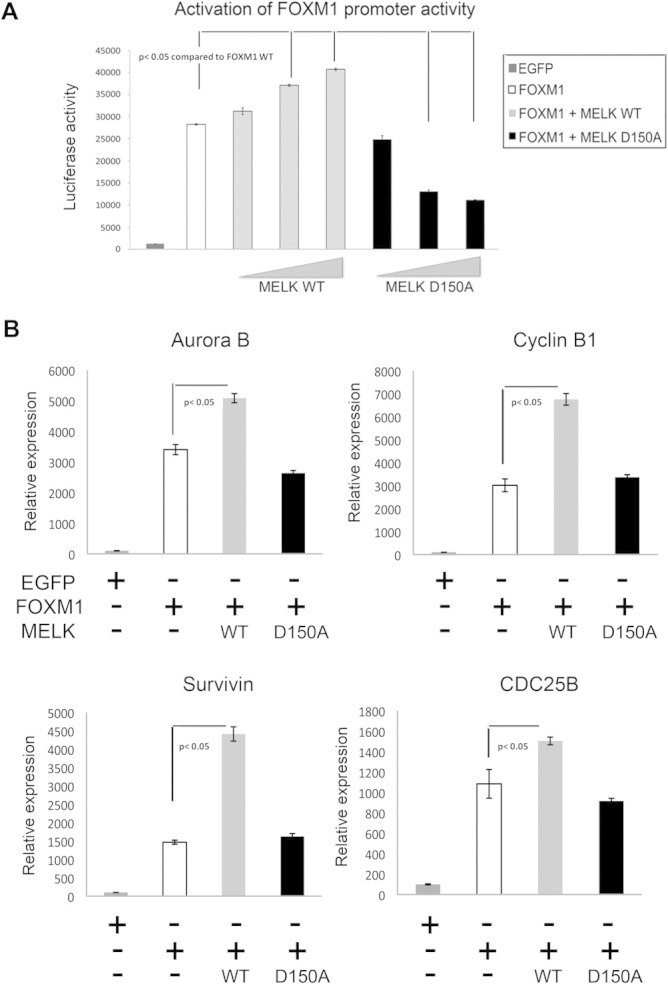
MELK phosphorylates FOXM1 and regulates *FOXM1* activity, leading to upregulation of mitotic gene expression. **(A):** Graph indicating the FOXM1 promoter activity in 293T cells transfected with the 6×*FOXM1* TATA-luciferase plasmid together with expression vectors encoding wild-type FOXM1 and increasing amounts of plasmids encoding either WT MELK or kinase-dead mutant form of MELK (D150A). The experiment was performed in triplicate in 96 well plates and repeated three times independently. **(B):** Relative mRNA expression levels of *Survivin, CyclinB1, CDC25B*, and *Aurora B* by quantitative reverse transcription polymerase chain reaction (qRT-PCR) in GBM30 cells transfected with GFP, FOXM1, FOXM1 + WT MELK, and FOXM1 + MELK D150A mutant. qRT-PCR was performed in triplicate and repeated three times independently. Abbreviations: EGFP, enhanced green fluorescent protein; WT, wild type.

To understand more in depth the molecular mechanism of MELK-dependent FOXM1 signaling, we investigated whether MELK regulates FOXM1 transcriptional activity using the 6× *FOXM1*-TATA-luciferase reporter plasmid. Transgene expression of wild-type FOXM1 alone activated its transcriptional activity, suggesting self-activation in GBM30 GSCs (Fig. 4A). Transgenic expression of wild-type MELK significantly enhanced *FOXM1* self-activation in a concentration-dependent manner (Fig. 4A). On the other hand, transgenic expression of the kinase dead mutant MELK D150A eliminated *FOXM1* self-activation in a concentration-dependent manner. Since FOXM1 is known to regulate the transcriptional network of genes essential for mitotic progression (e.g., *Aurora B kinase, CENPA, Survivin*, and *CyclinB1*), we examined whether the FOXM1-MELK complex had any effect on mitotic regulators in GBM30 GSCs. Cotransfection of FOXM1 with MELK resulted in a marked elevation of the key mitosis genes (Fig. 4B) [38]. In contrast, MELK D150A did not influence FOXM1-mediated mitotic transcriptional expression. Taken together, MELK kinase activity regulates phosphorylation and self-activation of *FOXM1*, leading to enhanced mitotic gene expression in GSCs.

### MELK-Driven Phosphorylation of FOXM1 Is PLK1 Dependant

**Figure 5:**
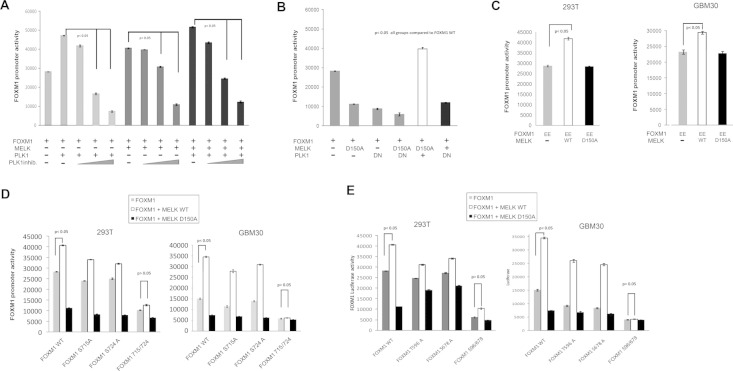
MELK-driven FOXM1 phosphorylation is dependent on PLK-1. **(A):** Graph indicating the *FOXM1* promoter activity in 293T cells with transfection of the FOXM1 reporter plasmid together with the plasmids encoding WT FOXM1 (FOXM1WT) and MELK WT, PLK-1 WT, or combination, in the presence of different doses (100, 250, and 500 nM) of the PLK-1 inhibitor ON-01910 for 48 hours. **(B):** Graph indicating the *FOXM1* promoter activity in 293T cells with transfection of the FOXM1 reporter plasmid together with the plasmids encoding either FOXM1WT and plasmids encoding for WT or kinase-dead mutant (D150A) of MELK or PLK-1 WT and DN PLK-1 mutant. **(C):** Graph indicating the *FOXM1* promoter activity in indicated cells with transfection of the FOXM1 reporter plasmid together with the plasmids encoding constitutive active mutant FOXM1 (FOXM1EE) and plasmids encoding for WT or kinase-dead mutant (D150A) of MELK. **(D):** Graph indicating the *FOXM1* promoter activity in indicated cells with transfection of the FOXM1 reporter plasmid together with the plasmids encoding either WT FOXM1 (FOXM1WT) or FOXM1 mutants (715A, 724A, or 715/724A) and plasmids encoding for WT or kinase-dead mutant (D150A) of MELK. **(E):** Graph indicating the *FOXM1* promoter activity in indicated cells with transfection of the FOXM1 reporter plasmid together with the plasmids encoding either WT FoxM1 (FOXM1WT) or FOXM1 mutants (S596A, S678A, or TSAA) and plasmids encoding for WT or kinase-dead mutant (D150A) of MELK. All luciferase experiments were performed using 96 well plate and repeated three times independently. Abbreviations: DN, dominant negative; GBM, glioblastoma multiforme; WT, wild type.

We then sought to determine the mechanisms that regulate *FOXM1* activation by MELK in GSCs. A recent study by Fu et al. [39] demonstrated that, similar to MELK, PLK1 binds and phosphorylates FOXM1 leading to *FOXM1* activation and increased gene expression required for mitotic progression in hepatocellular carcinoma cells. We therefore sought to dissociate the molecular signaling mechanisms of MELK-driven FOXM1 phosphorylation and PLK1-driven FOXM1 phosphorylation in GSCs. First, we used the PLK1 specific inhibitor ON01910 together with overexpression of FOXM1, MELK, and/or PLK1 and evaluated FOXM1 promoter activity (Fig. 5A). After confirming the elimination of PLK1-driven FOXM1 activation by ONO1910, we found that ONO1910 also eliminates FOXM1 activation driven by MELK in a dose-dependent manner (Fig. 5A). In order to exclude the possibility of an off-target effect by pharmacological inhibition, we then tested the effect of the PLK1 dominant negative mutant (PLK1 DN) on MELK-mediated *FOXM1* activation. Similar to the results with the pharmacological inhibition, forced expression of PLK1 DN eliminated MELK-driven *FOXM1* activation in GBM30 GSCs (Fig. 5B). To further confirm these results, we used the constitutive active form of FOXM1 (FOXM1 EE as PLK1-driven phosphomimics) to assess whether MELK D150 mutant is able to eliminate FOXM1EE-driven *FOXM1* activation in 293T and GBM30 GSCs (Fig. 5C). Unlike the MELK D150A-induced elimination of FOXM1 wild-type-driven self-activation, MELK D150A failed to diminish *FOXM1* activation driven by FOXM1 EE in both 293T cells and GBM30 GSCs. Taken together, these data suggest that MELK-regulated *FOXM1* activation is dependent on PLK1 in GSCs.

We next asked whether MELK-driven *FOXM1* activation is regulated by either the initial binding event of PLK1 to FOXM1 or the secondary trans-phosphorylation of FOXM1 following PLK1-FOXM1 binding. Specifically, Ser 715 and Ser 724 of the FOXM1 protein were mapped to be the major trans-phosphorylation sites and Thr-596 and Ser-678 were mapped to be the PLK1 binding sites [39]. A single mutation of Ser 715 or Ser 724 partially reduces the *FOXM1* self-activation, whereas a transgenic expression of the double mutation results in complete abolishment of *FOXM1* trans-activation [39]. Mutation of one of the two residues resulted in only a partial decrease in MELK-enhanced *FOXM1* self-activation in both 293T cells and GBM30 GSCs (Fig. 5D). On the other hand, introducing the double mutation led to a major reduction in MELK-dependent *FOXM1* transcriptional activity nearly to the level resulting from the loss of MELK kinase activity induced by the D150A mutation (Fig. 5D). We then performed the same experiments with the FOXM1 mutant vectors lacking the ability of PLK1 to bind FOXM1 either partially (single mutation vectors) or completely (double mutation vector) (Fig. 5E). Transgenic expression of the double mutation vector (FOXM1 596/678), but not the single mutation vectors (FOXM1 T596A and S678A), almost completely abrogated MELK-driven *FOXM1* activation both in 293T cells and GSCs. Taken together, these data indicate that the MELK-driven *FOXM1* activation is dependent on both the initial binding and subsequent trans-phosphorylation events by PLK1 in GSCs.

### Siomycin A Abrogates FOXM1-MELK Interaction

**Figure 6:**
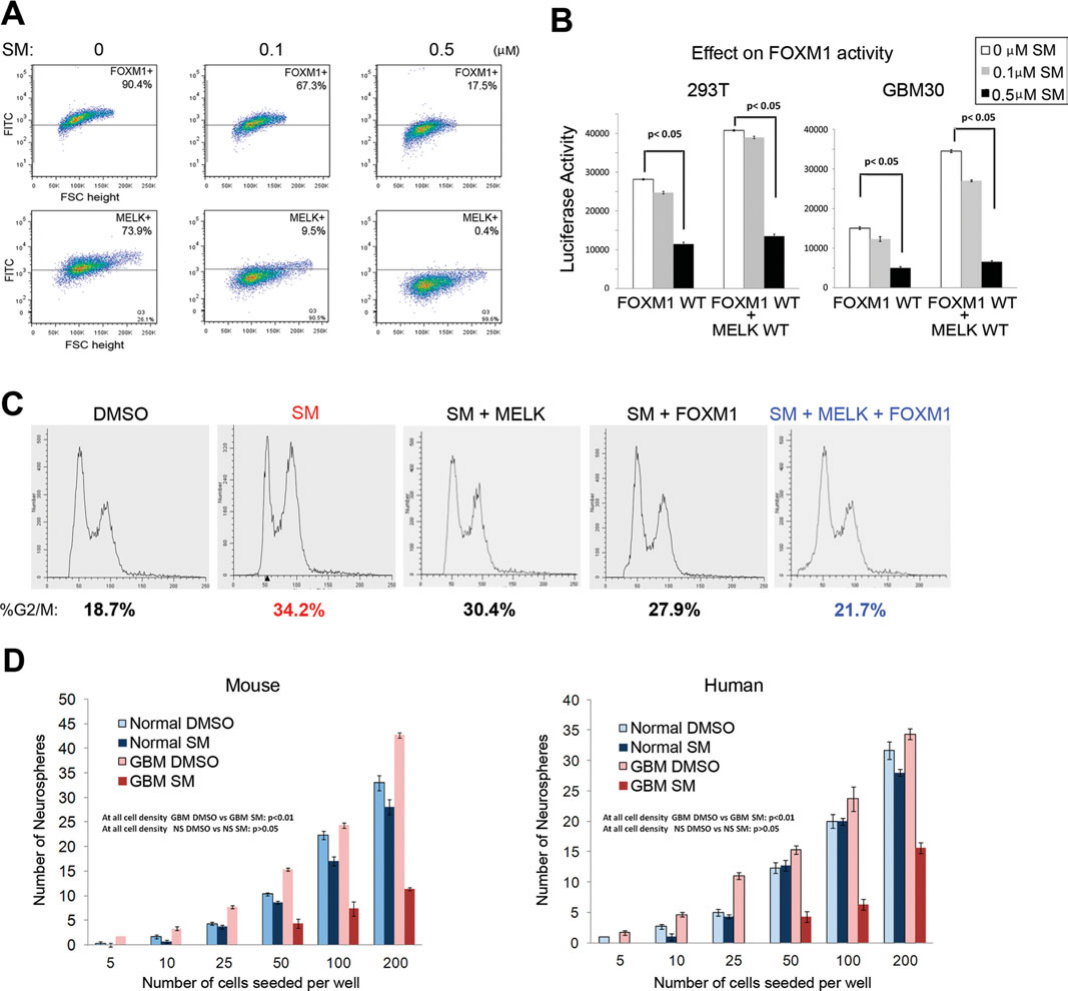
SM abrogates MELK-driven *FOXM1* activity and inhibits growth of glioma stem-like cells (GSCs) but not normal progenitors in mouse and human. **(A):** Flow cytometry analysis on GBM30 spheres treated with either DMSO or SM. The upper panels indicate the proportions of FOXM1 (+) cells in GBM30 spheres with indicated dose of SM treatment for 72 hours. The lower panels display the proportions of MELK (+) cells in each condition. Experiment was repeated three times and similar expression pattern was observed. **(B):** Graph indicating the *FOXM1* promoter activity in indicated cells transfected with the plasmids encoding for FOXM1 wild-type (WT) with MELK WT or kinase dead D150A. The cells were then treated with indicated doses of SM after transfection for 48 hours, followed by detection of *FOXM1* activity by luciferase assay. **(C):** Graph showing cell cycle analysis with flow cytometry of GBM30 GSCs. The cells were treated with either control (DMSO 1%), SM (500 nM), FOXM1 wild-type overexpression together with SM (500 nM), MELK wild-type overexpression together with SM (500 nM) or MELK, and FOXM1 wild-type overexpression together with SM (500 nM) for 48 hours. **(D):** Left panel indicates the relative neurosphere numbers formed from mouse subventricular zone or GBM-like tumors with SM (500 nM) treatment or DMSO. The right panel shows the relative neurosphere numbers formed from GBM30 GSCs and normal spheres (16wf).The different numbers of cells were seeded in each well, as shown on *x*-axis. DMSO concentration is 1% and SM was 500 nM. The experiments were done in triplicates using 96 well plates and repeated three times. Abbreviations: DMSO, dimethyl sulfoxide; FITC, fluorescein isothiocyanate; FSC, forward scatter; GBM, glioblastoma multiforme; SM, Siomycin A.

Previously, the thiazole antibiotic Siomycin A was identified as a FOXM1 inhibitor by diminishing its protein and mRNA abundance, and we recently discovered that Siomycin A also reduced MELK expression in GSCs in vitro [9, 18, 40]. Consistent with these findings, flow cytometry demonstrated that Siomycin A reduced the proportions of FOXM1 (+) and MELK (+) cells in GBM NS in a dose-dependent manner (Fig. 6A). We then investigated the effect of Siomycin A on *FOXM1* activity driven by FOXM1 and/or the FOXM1-MELK complex (Fig. 6B). We treated GBM30 GSCs with 0.1 μM or 0.5 μM of Siomycin A after transfection. Similar to the protein expression, *FOXM1* activity was almost abolished, declining to basal levels at 48 hours post-treatment with 0.5 μM of Siomycin A, suggesting that Siomycin A abrogates *FOXM1* activity driven by FOXM1 itself as well as MELK-driven FOXM1 phosphorylation. Next we evaluated whether restoring FOXM1 and MELK rescues Siomycin A-induced mitotic arrest in GSCs (Fig. 6C). In agreement with previous studies using other cancer cells, GSCs increased the proportion of cells in the G2/M phase when treated with 0.5 μM of Siomycin A (cells in G2/M phase: 18.72%–34.23%) [40, 41]. This data indicated that Siomycin A induces G2/M arrest of GSCs. Overexpression of FOXM1 with MELK, but not FOXM1 or MELK alone, rescued the Siomycin A-induced G2/M arrest of GBM30 GSCs (cells in G2/M phase: 34.23%–21.74%). These data provided supporting evidence for the critical role of the FOXM1-MELK complex in mitotic progression of GSCs.

Since FOXM1 levels were markedly higher in GSCs compare to NPCs (Fig. 2), we reasoned that the dependence of GSCs on FOXM1 would be greater than that of NPCs. Indeed, when we treated mouse and human GSC cultures (derived from Mut 6 mice and GBM30, respectively) and NPC cultures (derived from wild-type mice and a 16-week-old fetus-16wf, respectively) with varying doses of Siomycin A, GSCs derived from both species were 15-fold more sensitive to Siomycin A than NPCs, suggesting that pharmacologic inhibition of FOXM1 signaling by Siomycin A has stronger impact on in vitro growth of GSCs than NPCs (Fig. 6D).

### Combined Treatment of Temozolomide with Siomycin A on GSCs-Derived Mouse Tumors Yields Better Survival than Monotherapy with Temozolomide

**Figure 7:**
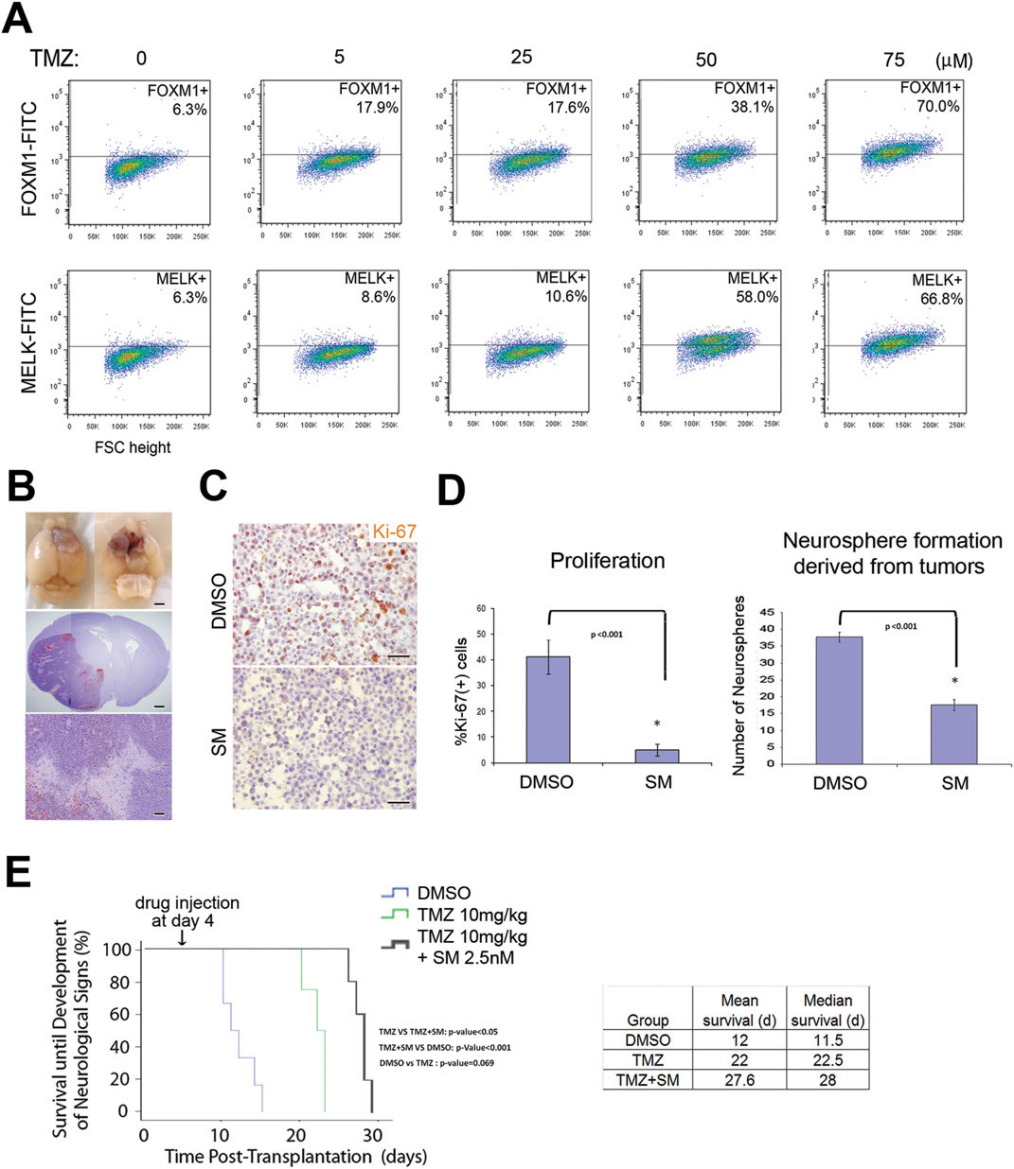
Combined treatment of TMZ with SM on glioma stem-like cell (GSC)-derived mouse tumors yields better survival than monotherapy with TMZ. **(A):** Flow cytometry analysis for FOXM1 (upper panels) and MELK (lower panels) with GBM30 SPGCs (serum-propagated GBM cells) treated with varying doses of TMZ for 72 hours. Experiment was repeated three times for confirmation of results. **(B):** Representative images of mouse brains with intracranial xenograft tumors derived from GBM30 neurospheres (left top panel). Middle and lower pictures indicate H&E staining. N indicates necrotic area in the tumor. Original magnifications: ×2 (middle panel) and ×10 (lower panel). Scale bar = 500 μm (middle panel) and 100 μm (lower panel). **(C):** Ki-67 immunohistochemistry of mouse tumors treated with either DMSO or SM. Mice were sacrificed at 2 days post-SM treatment. Original magnifications: ×20. Scale bars = 50 μm. Graph (right) indicating the proportion of Ki-67(+) cells in DMSO- and SM-injected tumors analyzed by Image J software. (*n* = 4 for each group) Asterisk (*) indicates statistical significance by *t* test. **(D):** Graph indicating the relative neurosphere numbers derived from mouse tumor tissues following DMSO and SM treatment for 2 days. (*n* = 3) Asterisk (*) indicates statistical significance by *t* test. **(E):** Kaplan Meier survival curve of mice harboring GBM30 neurosphere-derived tumors treated with DMSO (control), TMZ (10 mg/kg), or TMZ (10 mg/kg) combined with SM injection (2.5 nM). Table (right) indicates the mean and medial survival periods of the three groups. Abbreviations: DMSO, dimethyl sulfoxide; FITC, fluorescein isothiocyanate; FSC, forward scatter; SM, Siomycin A; TMZ, Temozolomide.

Accumulated evidence suggests that GSCs are relatively resistant to Temozolomide chemotherapy partly due to elevated expression of the drug resistance genes compared to non-GSCs [42–45]. We found that Temozolomide treatment of GBM30 GSCs paradoxically increases the expression of FOXM1 and MELK in a dose-dependent manner (Fig. 7A). These findings are consistent with the hypotheses that Temozolomide treatment spares FOXM1 and MELK expressing GSCs or promotes the appearance of a GSC-like phenotype.

We then investigated the effect of combination treatment with Temozolomide and Siomycin A on GSCs-derived mouse tumors in vivo (Fig. 7B–7E). Xenografting GBM30 NS resulted in the formation of GBM-like tumors in immunocompromised mouse brains. Similar to human GBM, the xenograft-derived tumors exhibited massive intratumoral hemorrhage, necrosis, and densely packed tumor cells with hyperchromatic nuclei surrounding tumor vessels (Fig. 7B). Immunohistochemistry of mouse tumors at 2 days post-Siomycin A injection demonstrated a significantly reduced fraction of Ki-67(+) proliferating cells compared to control-treated mouse tumors (Fig. 7C). In order to determine the effect of Siomycin A treatment on GSCs in vivo, we cultured NS forming cells of treated and control tumors. Tumors assayed at 2 days post-Siomycin A injection had significantly fewer NS-forming cells compared to the control tumors (Fig. 7D). These data suggest that Siomycin A has a potent inhibitory effect on survival and proliferation of GSCs in vivo. Subsequently, we assessed if Siomycin A treatment has any survival benefit on Temozolomide-treated mouse tumors. When the tumors were treated with Temozolomide alone, median survival of tumor-bearing mice was prolonged from 11.5 days to 22.5 days (Fig. 7E). The combined treatment with Temozolomide and Siomycin A exhibited further benefit on mouse survival (median survival to 28 days) (DMSO control vs. Temozolomide+Siomycin A: *p* < .001).

Finally, we used the tumor slice culture method to assess Siomycin A treatment on surgical GBM specimens [46]. Patient-derived GBM slice cultures were treated with DMSO, Temozolomide, or Siomycin A, and the effects were measured 16 hours post-treatment. H&E staining of the slice cultures demonstrated that the procedures did not destroy the cytoarchitecture of GBM tumors (Supporting Information Fig. S10). Immunohistochemistry with a proliferation marker, Ki67 demonstrated vast numbers of proliferating tumor cells in DMSO-treated samples, but not in Temozolomide- or Siomycin A-treated tissues (Supporting Information Fig. S11). In turn, immunohistochemistry for apoptosis marker activated Caspase-3 displayed significantly higher number of apoptotic cells in Siomycin A-treated samples, but not in DMSO- or Temozolomide-treated cultures (Supporting Information Fig. S11).

## DISCUSSION

Molecular signaling between protein kinases and transcription factors plays vital roles in tumor development and maintenance [47]. Here, we demonstrate the first evidence that the transcription factor/oncogene FOXM1 forms a protein complex with a serine/threonine kinase MELK and that FOXM1 serves as a substrate of MELK in cancer cells, and MELK-regulated FOXM1 phosphorylation controls *FOXM1* activity and induces the expression of downstream mitotic regulators. The transcription factor FOXM1 is a master regulator for cell cycle progression and is overexpressed in a number of human cancers including GBM [48]. The protein kinase MELK is also abundantly expressed in various cancers including GBM and plays a pivotal role in survival of cancer cells and cancer stem cells [12]. Recent studies have shed light on FOXM1 signaling in GBM and GSCs [10]. FOXM1 interacts with the promoter of the VEGF gene regulating its activation, which contributes to GBM tumor angiogenesis [49]. Another study by Zhang et al. elegantly showed that FOXM1 is a downstream component of canonical Wnt-signaling and directly binds to β-catenin, inducing its nuclear translocation and transcriptional activation [39]. This study demonstrates the first evidence that FOXM1 directly interacts and is phosphorylated by MELK in GSCs, further providing evidence for a role of FOXM1 signaling in GBM. Our data indicate that both phosphorylation of FOXM1 protein and transcriptional activity of FOXM1 depend on MELK kinase activity. Intriguingly, FOXM1 activation driven by MELK is dependent on both the priming event of the complex formation of FOXM1 with PLK1 and the subsequent trans-activation of FOXM1 by PLK1.

Despite the advances presented here, open questions still remain. First, it is not clear whether the MELK/FOXM1 protein complex plays a positive role in cancers or cancer stem cells in other organs. Second, it is undetermined whether MELK-mediated signaling is associated with the other proteins that regulate, or are regulated by, FOXM1 in cancer cells. Future studies are needed to elucidate these questions.

The cell of origin for GBM is still debatable, although the concept has been evolving. Here, we used a mouse model of gliomagenesis by ablation of key tumor suppressor genes in NPCs causing formation of GBM-like tumors in cerebral cortices. Our data indicated that FOXM1 signaling is operative in NPCs of the developing brains in vivo. Immunohistochemistry exhibited that abundance of FOXM1 protein is progressively elevated as cells progress from the neural progenitor stage through pretumorigenic progenitors to GSC. Sensitivity of GSCs to Siomycin A was markedly higher than that of NPCs. Taken together, these data indicate that GSCs are more dependent on FOXM1 signaling compared to NPCs. However, it remains unknown whether activation of FOXM1 signaling alone is sufficient for gliomagenesis and whether FOXM1 signaling plays a critical role in oligodendrocyte precursor-derived gliomagenesis. Future studies could help address these questions.

An important therapeutic implication of the present data is that combined therapy of mouse xenografted tumors with Temozolomide and Siomycin A resulted in diminished NS-forming GSCs in tumors and provided an additional benefit on mouse survival. Although eradication of cancer stem cells appears to be essential for the cure of cancers, recent studies also suggest that non-cancer stem cells acquire cancer stem cell phenotype when challenged by stressors such as ionizing radiation and chemotherapies [50]. To achieve cure of a cancer, eradication of the rare population of tumor cells that are considered as existing cancer stem cells may not be sufficient; instead, combination of non-cancer stem cell-targeted therapies and cancer stem cell-targeted therapies appears to be mandatory. Our data indicate that Temozolomide treatment increased, while Siomycin A decreased, both FOXM1 and MELK expression in GSCs. One interpretation of these data is the preferential eradication of non-stem tumor cells and subsequent enrichment of GSCs after treatment (selection of therapy-resistant tumor cell population; the clonal evolution theory). Alternatively, Temozolomide may induce phenotypic changes of the treated GBM cells and may increase FOXM1 and MELK expression (the molecular evolution theory). It is also possible that both theories are true. Future studies will address this open question.

## CONCLUSION

In this study, we demonstrated that the oncoprotein FOXM1 is regulated by the mitotic kinase MELK for its phosphorylation and autoactivation via a direct interaction in GSCs. Although FOXM1 is restrictedly expressed in NPCs in the normal brain and plays a role in NPC growth, a substantial elevation of FOXM1 during gliomagenesis and elevated sensitivity of GSCs over NPCs to pharmacological inhibition of FOXM1 signaling indicate that GSCs are more dependent on FOXM1 signaling for their survival and growth. Thus, targeting the ability of FOXM1 to form a protein complex with MELK may represent a potential therapeutic benefit for GBM. The results presented here may help to gain further insights into the biology of GSCs as well as molecular mechanism of tumorigenesis and therapy resistance in GBM.

## Supplementary material

Figure S1, related to Figure 1. FoxM1 immunohistochemistry. Immunohistochemistry for FoxM1 (green) and Tuj1 (red) in mouse brains of embryonic day 17.

Figure S2, related to Figure 1. FoxM1 regulates neural progenitor proliferation. (A)Quantitative RT-PCR analysis of FoxM1b in E17 neurospheres overexpressing FoxM1 (isoforms b) or EGFP (control) (N=3). (B) Left panel representing FoxM1 mRNA expression in E17 neurospheres after transfection with siRNA for either mock, FoxM1a, or FoxM1a/b. The numbers indicate the cycles of PCR reaction. Gapdh is used as internal control. Right panel shows picture representing siRNA treated spheres. Original magnification 10X. (C) Neurosphere forming assay with clonal density of E17 neurospheres overexpressing FoxM1 (isoforms b) or EGFP (control). Neurosphere forming assay were performed in triplicates in 96 well plate and repeated at least three times independently. Scale bars represent 100 micron. (D) Neurosphere forming assay with clonal density of E17 neurospheres after transfection with siRNA for either mock, FoxM1a, or FoxM1a/b. Neurosphere forming assay were performed in triplicates in 96 well plate and repeated at least three times independently.

Figure S3, related to Figure 2. FOXM1 in mouse GBM model. FoxM1 immunohistochemistry in non-tumor regions of Mut6 mice. Hematoxylin counterstaining is shown in blue. Original magnifications, 20X. Scale bars represent 50 micron.

Figure S4, related to Figure 2. FOXM1 in human GBM tumors. FOXM1 staining with human GBM tissues, showing nuclear FOXM1 immunoreactivity. Blue staining indicates Hematoxylin counterstaining. Original magnifications, 20X. Scale bars represent 50 micron. Similar staining pattern was observed in 5 different patient samples.

Figure S5, related to Figure 2. FOXM1 expression in GSCs. (A) Graph (upper panel) indicating the relative FOXM1 mRNA expression determined by qRT-PCR in undifferentiated GBM1600 neurospheres (GSCs) and their sister cultures in pro-differentiation condition (SPGCs). (B) Histograph (lower panel) indicating the relative FOXM1 protein expression determined by flow cytometry with the same sample set described above.

Figure S6, related to Figure 2: FOXM1 activity in GSCs. Graph indicating the FOXM1 promoter activity in GBM30 GSC vs. SPGCs cells with transfection of the 6×FOXM1 TATA-luciferase plasmid.

Figure S7, related to Figure 3. Correlation of FOXM1 and MELK expression. Evaluation of correlation of MELK and FOXM1 mRNA expression profile (Affymetrix Human Genome U133A Array) with newly-diagnosed GBM (A) (n=58) and recurrent GBM (B)(n=22).

Figure S8, related to Figure 3. Transcriptional interaction between FOXM1 and MELK. (A) RT-PCR demonstrating the effect of overexpression of FOXM1, MELK, or GFP (control) on mRNA expression of FOXM1 and MELK. GAPDH is used as internal control. (N=3)

Figure S9, related to Figure 3. Abundance of GFP protein in MELK immunoprecipitated HEK 293 cells after transfection of the plasmids encoding Empty-Flag, MELK-Flag, and MELK D150A-Flag.

Figure S10, related to Figure 7. Slice cultures of GBM surgical tissues. Representative H&E staining of a slice culture of GBM surgical tissue treated with DMSO for 72 hours, exhibiting the preservation of the cytoarchitecture of tumors in this assay. Original magnifications, 10X Scale bar represents 100 micron.

Figure S11, related to Figure 7. Effect of Siomycin A on GBM slice cultures. Effect of Temozolomide (TMZ) or Siomycin A (SM) treatment on cell survival and proliferation using the slice cultures of GBM surgical tissues. Experiment was repeated suing four different patient samples.
