# Supplementary figures and images for: MELK-Dependent FOXM1 Phosphorylation is Essential for Proliferation of Glioma Stem Cells
Source: Stem Cells. 2013 Feb 13;31(6):1051–63. doi: 10.1002/stem.1358 (PMC3744761; doi:10.1002/stem.1358)

**TuJ1**

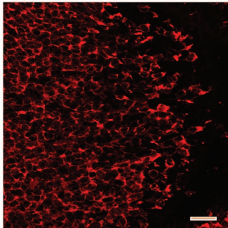

**FoxM1**

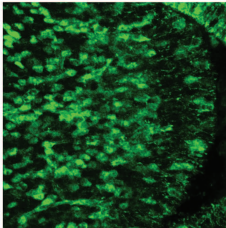

**Overlay**

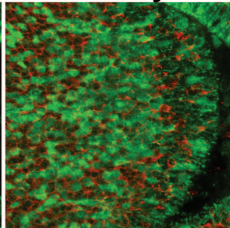

Supplement: Supplementary file 1 [file stem0031-1051-SD1.pdf]

# FoxM1 Expression in Mut6 Brain

Leptomeninges

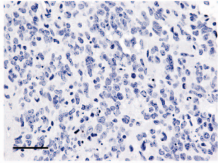

Midbrain

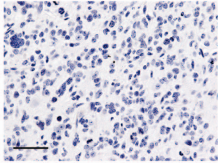

Cerebellum

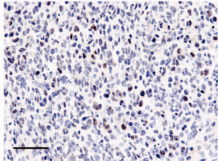

Supplement: Supplementary file 3 [file stem0031-1051-SD3.pdf]

**A**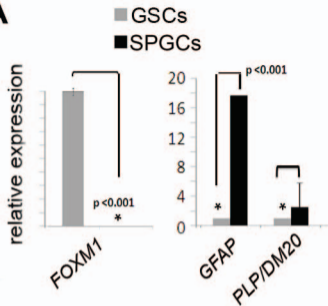**B**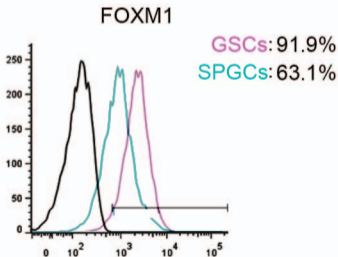

Supplement: Supplementary file 5 [file stem0031-1051-SD5.pdf]

## FOXM1 promoter activity

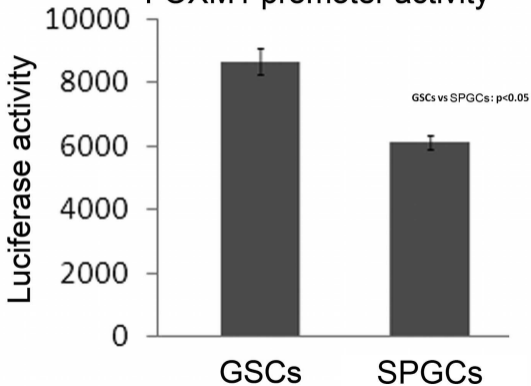

Supplement: Supplementary file 6 [file stem0031-1051-SD6.pdf]

**A** Newly-diagnosed GBM

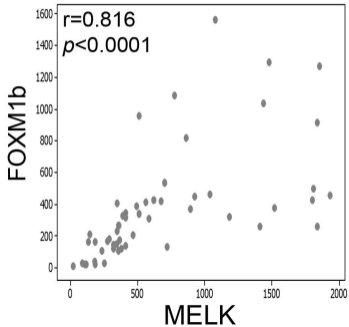

**B** Recurrent GBM

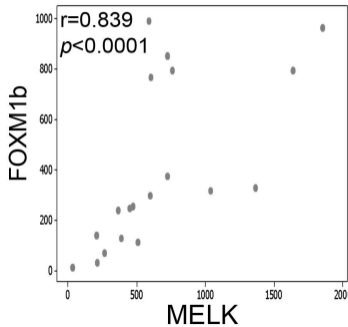

Supplement: Supplementary file 7 [file stem0031-1051-SD7.pdf]

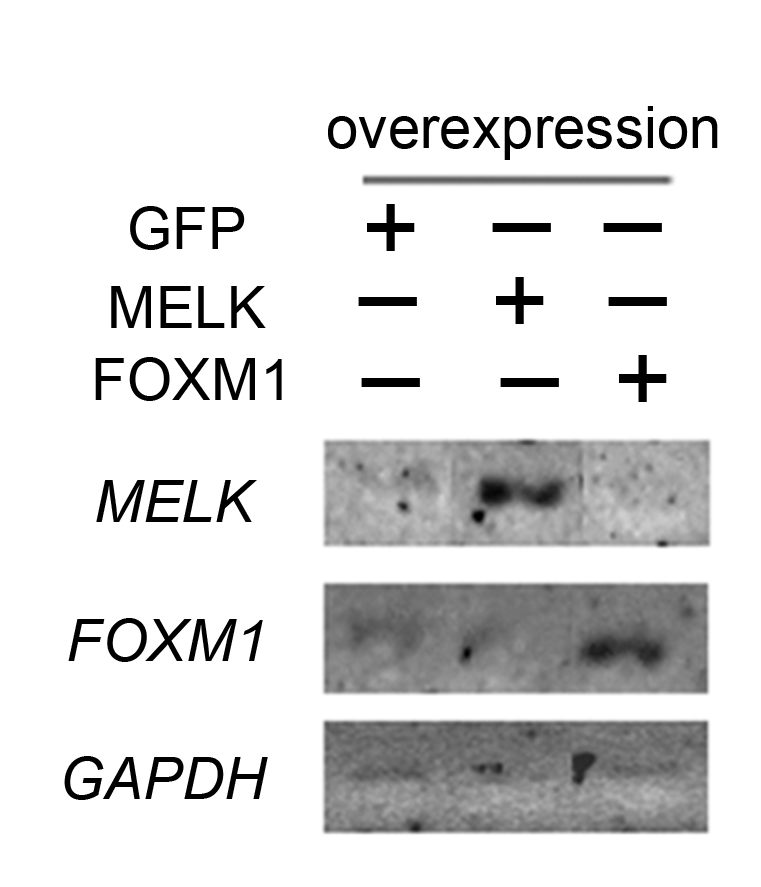

Supplement: Supplementary file 8 [file stem0031-1051-SD8.tif]

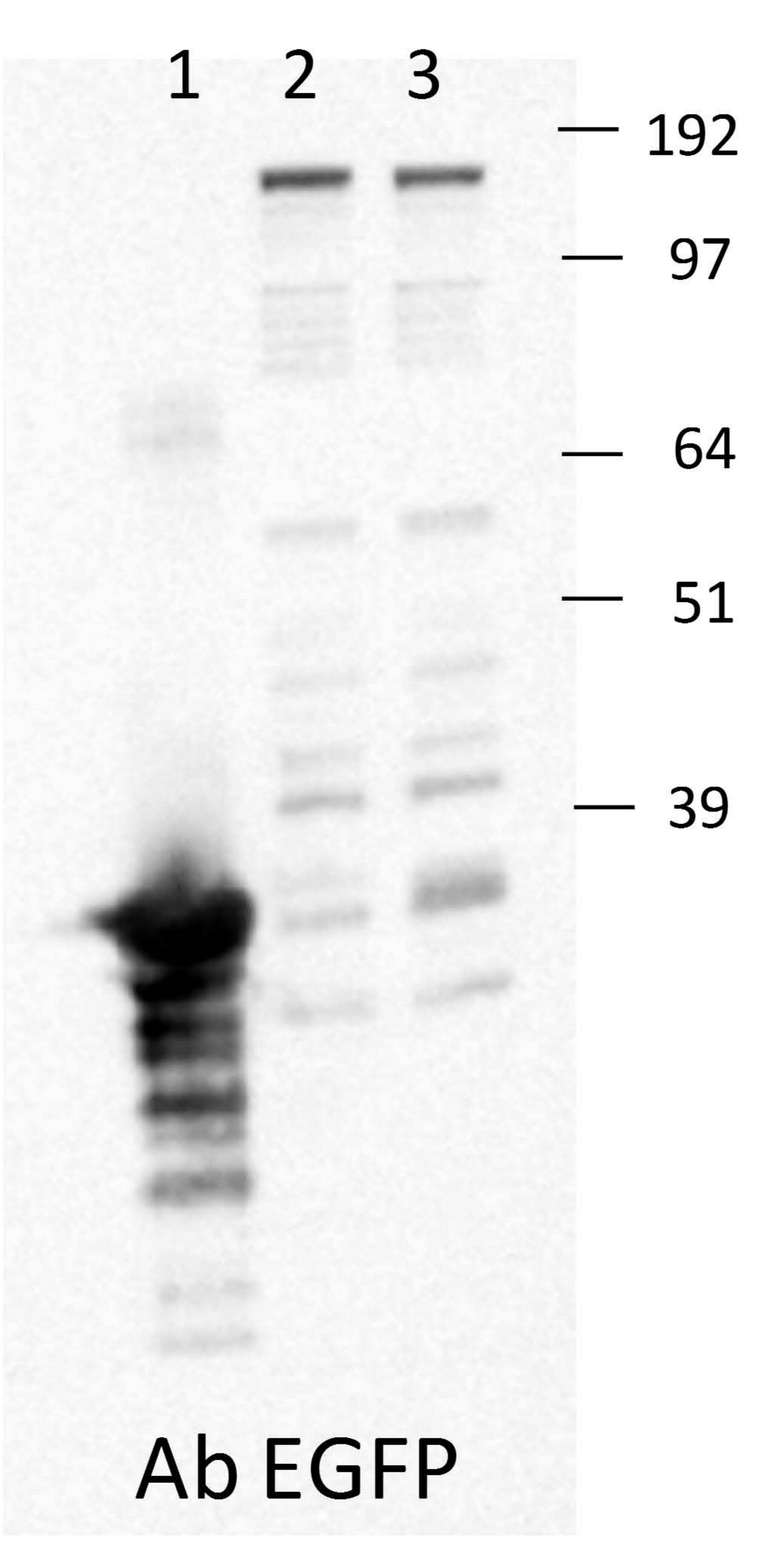

Supplement: Supplementary file 9 [file stem0031-1051-SD9.pdf]

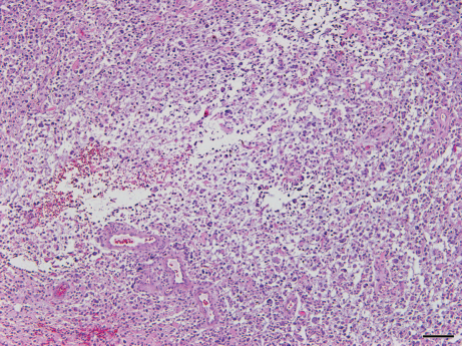

Supplement: Supplementary file 10 [file stem0031-1051-SD10.pdf]

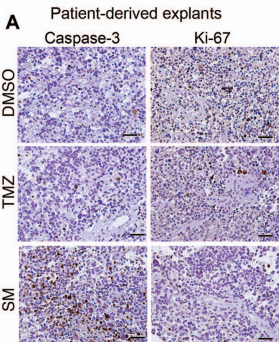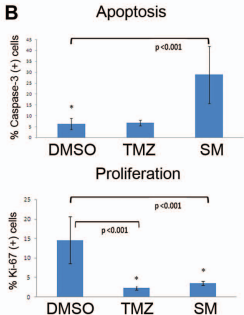

Supplement: Supplementary file 11 [file stem0031-1051-SD11.pdf]
