# Supplementary material for: MELK-Dependent FOXM1 Phosphorylation is Essential for Proliferation of Glioma Stem Cells
Source: Stem Cells. 2013 Feb 13;31(6):1051–63. doi: 10.1002/stem.1358 (PMC3744761; doi:10.1002/stem.1358)
Supplement: Supplementary file 2 [file stem0031-1051-SD2.pdf]

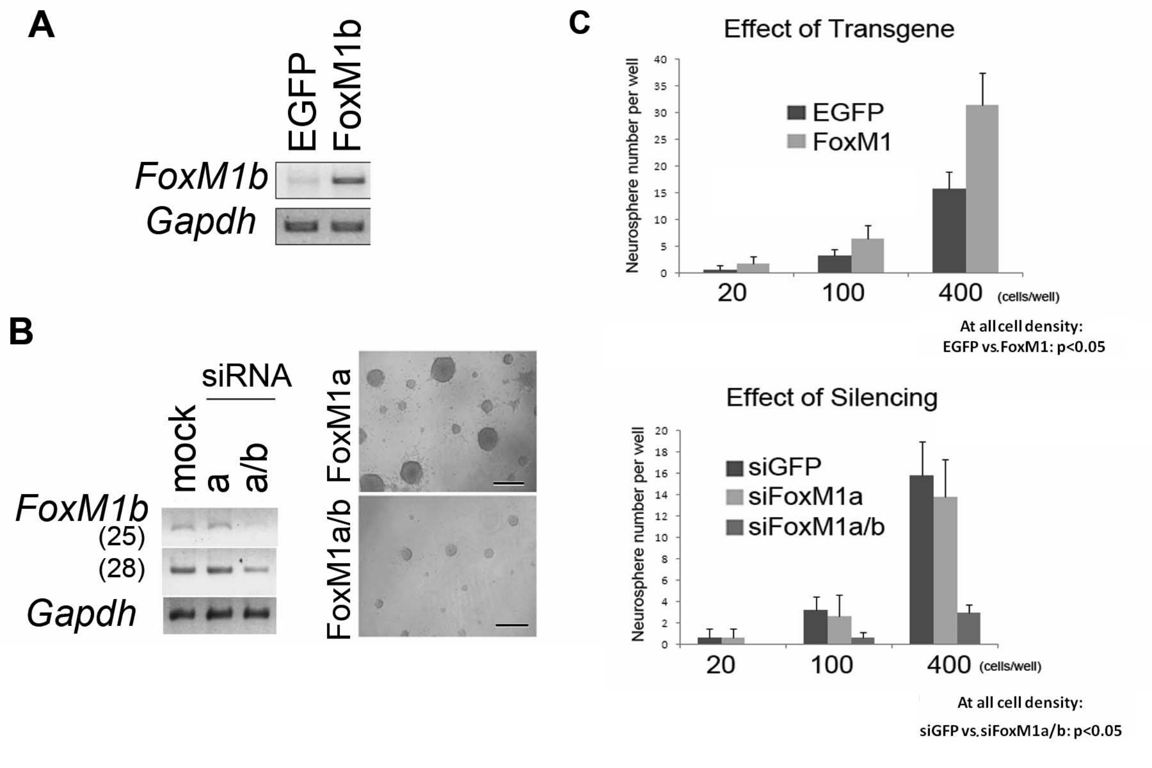
A
EGFP
FoxM1b
FoxM1b
Gapdh
B
siRNA
mock
a
a/b
FoxM1b
(25)
(28)
Gapdh
FoxM1a
FoxM1a/b
C
Effect of Transgene
Neurosphere number per well
EGFP
FoxM1
20
100
400
(cells/well)
At all cell density:
EGFP vs.FoxM1: p<0.05
Effect of Silencing
Neurosphere number per well
siGFP
siFoxM1a
siFoxM1a/b
20
100
400
(cells/well)
At all cell density:
siGFP vs.siFoxM1a/b: p<0.05
