# Supplementary material for: MELK-Dependent FOXM1 Phosphorylation is Essential for Proliferation of Glioma Stem Cells
Source: Stem Cells. 2013 Feb 13;31(6):1051–63. doi: 10.1002/stem.1358 (PMC3744761; doi:10.1002/stem.1358)
Supplement: Supplementary file 12 [file stem0031-1051-SD12.pdf]

SUPPLEMENTAL INFORMATION

MELK-dependent FOXM1 phosphorylation is essential for proliferation of glioma stem cells.

Kaushal Joshi[1], Yeshavanth Banasavadi-Siddegowda[1], Xiaokui Mo[2],Sung-hak Kim[1], Ping Mao[1], Cenk Kig[3], Diana Nardini[5,6] , Robert W. Sobol[7], Lionel M.L. Chow[5,6], Harley I. Kornblum[3], Ronald Waclaw[5,6], Monique Beullens[4], and Ichiro Nakano[1]*

SUPPLEMENTAL MATERIALS AND METHODS

Human specimens and tissue culture. Primary human glioma stem like cell lines GBM84, GBM 528, GBM 718, GBM 816, GBM 1600 1123, AC17, AC20 were obtained from freshly resected human malignant glioma specimens (GBM 30,1384, 528, 718, 816, 1600 are Grade IV glioblastoma and 1123, AC17 and 20 are Grade III oligodendrocytoma). Normal stem cells were isolated from 16 week fetus (16WF) brain. Human neural stem cells were reported previously. Neurospheres were cultured in DMEM/F12 supplemented with bFGF (Peprotech), EGF (Peprotech), B27(Invitrogen), heparin (Sigma-Aldrich), penicillin/streptomycin (Invitrogen), and L-glutamine (Invitrogen).[16-17] For neurosphere-formation assay, cells were dissociated into single cells and plated into 96-well plates in a 0.1 ml volume of growth media using serial dilutions, and the resultant neurosphere numbers were counted at 7-14 days. 293T cells were obtained from the American Type Culture Collection. Mouse sub-ventricular zone (SVZ) cells (mouse neural stem cells-NSCs) were obtained by harvesting mouse brain after euthanasia, isolating SVZ region, dissociating tissue with Tryple Express

(Invitrogen) and culturing as neurospheres in stem cell medium. For differentiation, the GBM samples labeled SPGCs (serum propagated glioma cells) were cultured in DMEM/ F12 (Invitrogen) with 10% FBS (Invitrogen), and were passaged when the culture was confluent. SPGCs are progeny of GBM spheres that was cultured in serum-containing medium with minimal to no CD133 expression. To generate neurosphere from mouse bearing human GBM30 tumors, mice were sacrificed when symptomatic. The mouse brain bearing human tumors were dissociated using scalpel and incubated with TrypLE™ Express (Invitrogen). The dissociated tumors were washed with PBS and grown as neurosphere in serum free medium containing EGF and bFGF.

Immunocytochemistry and Immunohistochemistry*.* Immunocytochemistry and immunohistochemistry were performed as described previously. [12,16,17] We used following primary antibodies: FOXM1 (Santa Cruz-k-19), Nestin (Millipore), SOX2 (Millipore), MELK (sigma-aldrich) , GFAP (Sigma) , TuJ-1 antibody (Covance and Berkely antibodies), BrdU antibody (ABCAM), Sox2 antibody rbt-polyclonal (Seven Hills Bioreagents), DCX antibody GP-polyclonal (Millipore). Primary antibodies were visualized with Alexa Fluor 555, 488, conjugated secondary antibodies (cell signaling), Donkey anti-Rat, Donkey anti-Rabbit, both conjugated to Cy3. Hoechst 33342 (blue) was used as a fluorescent nuclear counterstain. In all experiments, specific labeling of the antibodies was confirmed by the negative control samples without primary antibodies. For immunohistochemistry, mice were perfused transcardially with ice-cold PBS followed by ice-cold 4% paraformaldehyde in PBS, pH 7.4. Brains were removed, fixed in 4% paraformaldehyde overnight, equilibrated in 20% sucrose PBS, frozen in dry

ice, and stored at −80°C until use. Sections (20 μm) were cut on a cryostat and were incubated overnight at 4°C in the presence of 0.25% Triton, and 10 % normal goat serum and the primary antibody. The Envision system (Dako) using Diaminobenzidine (DAB) as a chromogen was used for detection of primary antibody following the manufacturer's protocol. Hematoxylin or Hoechst 33342 were used as a nuclear counter stain. Our entire mouse FoxM1 staining was done using a Tyramide Kit (Invitrogen). For BrdU staining P30 mice were injected (i.p.) with 100mg/kg of BrdU (sigma) and sacrificed after 2 hours.

Cell growth assay. GBM neurospheres were dissociated into single cells. The numbers of live cells were counted with hematocytometer after Trypan Blue (Gibco) staining and 1,000 cells were seeded into each well of 96-well plates that contain 100μL culture medium. After treatment, the cell number of each well was estimated using Alamar Blue. (Invitrogen) following the manufacturer's protocol. Assays were performed in triplicates and repeated three times independently.[12,16]

Xenograft. All the animal experiments were approved by Institutional Animal Care and Use Committee following NIH guidelines using athymic nu/nu mice (NCI/NIH). On the day of transplantation, GBM30 spheres were dissociated using TrypLExpress (Invitrogen) followed by trituration, washed, and total live cells was estimated using Trypan Blue exclusion. Cells were resuspended in phosphate-buffered saline (PBS) at a density of 2000 live cells/μL and stored at 4°C until injection. Animals were anesthetized using 50mg/Kg of Ketamine and 25mg/Kg of Xylazine. A total of 10000

cells were injected over 5 minutes into the brain (neostriatum) using the coordinates of 0.5 mm anterior from Bregma, 2 mm lateral from the midline, and 3.0 mm below the pial surface. 5 µL solutions containing SiomycinA (2.5 nmol) or DMSO diluted in PBS were injected into the same location with the same procedure at day 4. We used 7 mice in DMSO group, 4 mice in TMZ only and 5 mice in TMZ+SM group for xenograft experiment Animals were sacrificed if symptomatic. [12, 16, 17]

Flow cytometry analysis. Flow cytometry was performed as described previously.[24] GSC were collected, dissociated into single cells by using TrippleExpress and fixed by 4% paraformaldehyde. After blocking with 10% NGS in 0.1% triton/1XPBS cells, GSC were incubated with FOXM1 antibody (santa Cruz) for 1 hour and Alexa flour 555 (red) for 1 hour at 4 °C, followed by analysis with flow cytometry using a FACS Calibur flow cytometer (BD Biosciences). Cell cycle analysis was performed using Vybrant® DyeCycle™Ruby Stain (Invitrogen- CA). The data were analyzed by FlowJo 7.6.1 software. For CD133 cell sorting, the cells were stained using CD133 cell stain kit (Miltenyi- Biotech) and cells were sorted using BD-FACS-ARIA-II instrument at Flow cytometry core facility at the Ohio State University. Cell Pellets were collected right after sorting and washed with PBS. The RNA was isolated from the cell pellet according to manufacturers (Qiagen) protocol.

Luciferase assays. HEC293T cells were transfected with the indicated plasmids using Lipofectamine 2000 according to manufacturer’s protocol. Luciferase activity was

determined 48 hours after transfection using Luciferase Assay System (Promega). Experiments were performed in triplicate.

RNA Isolation. Cell pellet were lysed with 1ml Qiazol Lysis Reagent. Total RNA was then extracted and purified using the Qiagen miRNeasy Mini kit according to the manufacturer's instructions. Briefly, after mixing with 200µl chloroform, each cell lysate was centrifuged at 12,000xg to separate the aqueous and organic phases. Each aqueous phase was transferred to a clean 1.5 ml tube and then mixed with 1.5 volumes 100% ethanol. The mixture was then transferred to an RNeasy Mini column and RNA was bound to the capture membrane by a 30 second centrifugation at 12000 rpm. After one wash with buffer RWT and then two washes with buffer RPE, RNA was eluted from each column with 30µl RNase-free water. For each cell cultures, three independent RNAs were prepared. In all cases, the average RNA integrity number (RIN) was greater than 9.0. RNA concentration was determined using a Nanodrop 2000.

Microarray analysis. Comparative analysis of mRNA expression was performed using the Human U219 Array Strip and the Affymetrix GeneAtlas system, as per the manufacturer's instructions. Microarray analysis for each of the cell cultures (in triplicate) was accomplished with 100ng purified total RNA (described above) as the initiate material and the corresponding amplified and labeled antisense RNA(aRNA) using an GeneCHip 3'IVT Express kit (Affymetrix), as described by the manufacturer. The resulting aRNA was fragmented and end-labelled, as described by the manufacturer. The labeled aRNAs were then mixed with hybridization master mix and

the hybridization cocktails were then denatured at 95°C for 5 minutes, followed by 45°C for 5 minutes then kept at 45°C until applied to the hybridization tray (GeneAtlas System; 120µl hybridization cocktail of a cell culture was transferred into a well of a 4 well hybridization tray). The array strip was immerse into hybridization cocktail and incubated in the Hybridization Station at 45°C for 16 hours. After hybridization, the strip was washed and stained in the GeneAtlas Fluidics Station using the GeneAtlas Hybridization, Wash, and Stain Kit (Affymetrix #900720) and the intensity of each hybridized probe was generated using the GeneAtlas™ Imaging Station. Raw .cel files from the Human U219 Array Strip were analyzed using the 'oligo' package in R Bioconductor specifically designed to analyze Human U219 arrays. The raw data was normalized and summarized using robust multichip average (RMA). The data summarized by transcript clusters was used for further analysis. For transcripts represented by multiple clusters, the cluster with the highest IQR (Interquartile range; a descriptive statistic used to summarize the extent of the spread of the data) was selected to represent the transcript's expression. As a result of the filtering procedure, all transcripts are represented by a single cluster for further statistical analysis.

The 'genefilter" package in R (Bioconductor.org) was used to perform differential gene expression analysis. Genes differentially expressed in Proneurol versus Mesencheymal cell cultures were identified using the univariate t test ($p < 0.001$). The p-values obtained were adjusted for multiple comparisons using the method of Benjamini and Hochberg.

Quantitative and conventional RT-PCR. Transient transfection. RNA isolation, cDNA synthesis, and RT-PCR were performed as described. [19,24]Transfection of siRNA for

mouse spheres were performed with Lipofectamine 2000 (Invitrogen) according to the manufacturer's protocol as described previously. Sequences of primers were as follows:

FOXM1:

sense, 5'- TAGGGTGGCCTCTCAGACAC-3',

antisense, 5'- AGCGTTAAGCAGGAACTGGA-3',

NFH:

sense,5'-CCACGGAAAAGCCCAAGGACTCTA-3',

antisense, 5'-GGGGGCTGGCTTTCTTTCTGG-3',

MELK:

sense,5'-CTTGGATCAGAGGCAGATGTTTGGAG-3',

antisense, 5'-GCTGTAATCTTGCATGACCCAGG-3',

GFAP:

sense,5'-GCAGAGATGATGGAGCTCAATGACC-3',

antisense, 5'-ACCTCCAGCCGGGCACTG-3',

Survivin:

sense, 5'- CTTTCTCCGCAGTTTCCTCA -3',

antisense, 5'- TTGGTGAATTTTTGAAACTGGA-3'.

Aurora B:

sense, 5'- CGAATGACAGTAAGACAGGGC-3',

antisense, 5'- TGCACCACTTGGAACAGTTT-3'.

CDC25B:

sense, 5'- GCCTGTTCAAGTTCTCTGGC-3',

antisense, 5'- CCTAGGAGAAGACCAGGCAG-3'.

CyclinB1:

sense, 5’- ACAGGTCTTCTTCTGCAGGG-3’,

antisense, 5’- GAACCTGAGCCAGAACCTGA-3’.

GAPDH:

sense, 5’-CGGAGTCAACGGATTTGGTCGTAT-3’,

antisense, 5’-AGCCTTCTCCATGGTGGTGAAGAC-3’.
